# Construction process and development stages of pandemic emotions questionnaire in cancer patients (PEQ-CP)

**DOI:** 10.1186/s40359-022-00930-5

**Published:** 2022-09-27

**Authors:** Laura Gangeri, Sara Alfieri, Margherita Greco, Marco Bosisio, Rossella Petrigliano, Luciana Murru, Claudia Borreani

**Affiliations:** Clinical Psychology Department, Fondazione IRCCS Istituto Nazionale Dei Tumori, Via Venezian 1, 20133 Milan, Italy

**Keywords:** CoViD, Cancer, Emotions, Mixed method, Psycho-oncology, Questionnaire, Pandemic

## Abstract

**Background:**

Despite the numerous tools built ad hoc to investigate the effects of the CoViD-19 pandemic on people, to date there are no known questionnaires that investigate the emotional experiences of cancer patients. This work aims to start a process of construction and validation of a tool that captures these aspects (Pandemic Emotions Questionnaire in Cancer Patients—PEQ-CP).

**Method:**

A mixed method approach was used through three phases, each on a different sample: Phase 1: creation of items and checking of internal validity, through unstructured interviews and verification of the validity of content by "peers" and "experts"; Phase 2: exploration of the factorial structure of the scale through an exploratory factor analysis (EFA); Phase 3: confirmation of the factorial structure of the scale through a confirmatory factor analysis (CFA).

**Results:**

Phase 1 revealed 26 items that can be grouped into 4 theoretical dimensions. "Peers" and "experts" rated all items as understandable and relevant except one, which was reformulated. The EFA in the Phase 2 revealed a factorial structure with 14 items and three dimensions (Emotional Understanding, Communication of Emotions, Feelings the same as others), confirmed by the CFA in Phase 3.

**Conclusion:**

Although further validation steps are required, the PEQ-CP showed good psychometric properties.

**Supplementary Information:**

The online version contains supplementary material available at 10.1186/s40359-022-00930-5.

## Background

On January 30, 2020, the World Health Organization declared the CoViD–19 epidemic a public health emergency of international interest [1, 2]. The CoViD-19 pandemic spread rapidly around the world, affecting many people in Italy as well. The national and international health systems have made many efforts to contain infections and emergencies of infected patients, with the aim of providing adequate medical care during this period and ensuring the care of patients with other diseases.

The restrictions adopted to contain the spread of the virus (quarantine, lockdown, etc.) seem to have had an impact on the physical and psychological health of millions of people. Some researches have found increased psychological distress during the early stage of the CoViD-19 outbreak in terms of anxiety, depression, and post-traumatic symptoms ranging from moderate to severe levels [3–6].

Some studies have investigated the psychological impact on people who have contracted CoViD-19. They report that over 90% of clinically stable patients reported significant post-traumatic stress symptoms, especially when associated with certain variables such as older age, the presence of physical symptoms, lower education levels [5, 7–10].

Other studies have focused on health care workers, noting increased stress, anxiety, depression and sleep disturbances particularly in women, younger staff, and the nursing profession [11–15].

Less attention has been given to the reality of cancer patients, despite the unfavorable impact of CoViD-19 on health and economic systems around the world, which has caused disruption of treatment pathways and screening interventions due to the necessary measures to minimize exposure of patients to the virus [12, 16, 17].

A systematic review of the literature that collected 1110 studies from 10 countries around the world describes the situation of cancer patients highlighting some issues that emerged during the pandemic: non-participation in screening paths, delays or postponements of cancer treatment programs, diagnostic delays, drug shortages, inadequate nursing care, reduction of psychological support paths [18]. These factors seem to cause an increase in anxiety, a sense of loneliness and abandonment, also due to the lack of family members within the hospitals [18–22]. The sense of loneliness, the experience of isolation and the lack of moments of distraction related to little or no social life also seem to fuel concerns about cancer and a process of focusing on thoughts related to the disease [19].

At the same time, isolation seems to be associated with a moment/opportunity to reflect on oneself, on the condition of illness with a vision also on the positive and growth aspects. Research has shown that cancer patients forced home by the pandemic reported to have experienced a sense of belonging to the rest of the community that was equally in isolation [23]. Arrato et al. [24] suggest that patients with cancer may be more resilient to CoViD-19 stressors than has been assumed. The results of their study showed that lung cancer patients have significantly less stress, less worry about their family contracting the virus, greater success with social distancing and having few psychological symptoms compared to a control group. The authors concluded that these patients showed resilience. Resilience can be defined as “a dynamic process of positive adaptation in the context of significant adversity” [25] (p. 858).

A resilient attitude of cancer patients was also observed in clinical practice within our Comprehensive Cancer Center (Fondazione IRCCS Istituto Nazionale dei Tumori, Milan). It is an experience that contains different elements from those documented up to now in the general population, in operators and in CoViD-19 patients. In clinical practice, the state of psychological well-being emerges in particular from the experience of feeling more understood than from the fears of illness and uncertainties about the future. Moreover it emerges a general feeling of being "in the same boat" or more similar to everyone else. These elements suggest that we can adopt the salutogenic approach as the theoretical framework. Salutogenic approach is a social sciences approach focusing on the study of the origins of health (and not of the origins of disease) that seek better understanding the positive aspects of human experience [26, 27].

These contents are innovative with respect to the variables covered by the tools currently present in the literature [28, 29]. The two tools that investigate the impact of CoViD-19, the Fear of CoViD-19 Scale (FCV-19S) [28] and the Coronavirus Anxiety Scale (CAS) [29] focus on the evaluation of aspects related to psychological distress, such as anxiety and fear and they don’t include elements relating to adaptability, resilience and well-being, not using a salutogenic approach.

The present study aims to begin a process of construction and validation of a questionnaire that investigates the emotional perceptions of cancer patients during a pandemic, that we called Pandemic Emotions Questionnaire in Cancer Patients (PEQ-CP).

To achieve this aim, the study involved three phases, using a mixed method approach:

1. Creation of items and checking of content validity (phase I)
2. Exploration of the factorial structure of PEQ-CP (phase II)
3. Confirmation of the factorial structure of the PEQ-CP (phase III)

This project was approved by the institutional review board (Fondazione IRCCS Istituto Nazionale dei Tumori of Milan - INT 189/20).

The study began in February 2020 and ended in October 2021, going through various stages of the pandemic. Additional file 1: Appendix 1 shows the chronogram of the research phases in the development of the pandemic.

## Phase I

Phase I aims to create a list of items regarding the patients emotional experience during the CoViD-19 pandemic. The rationale for Phase I is to try to obtain a list as complete as possible of statements regarding emotional experience.

### Method

#### Procedures

In order to reach the research aim of Phase I, the procedure suggested by Chiorri [30] for the construction of new tools was implemented. This procedure requires (1) the creation of a series of items starting from textual (qualitative) material; (2) the involvement of "peers" (in our case, cancer patients) and "experts" (in our case, professionals working with cancer patients and psychometrists) in evaluating content validity of these items.

In February 2020, psycho-oncologist in the Psychology Department of  Fondazione IRCCS Istituto Nazionale dei Tumori began to notice some characteristic emotions of the way cancer patients were experiencing the pandemic. Starting from this clinical evidence, three unstructured interviews were conducted with three cancer patients, who were asked to tell their emotional experiences during the pandemic through the following question: "In emotional terms, how are you living this moment of pandemic?". Subsequent questions in line with the aim were asked following the respondents' discursive flow.

Starting from the statements that emerged from these three interviews and from the observations collected by clinical psycho-oncologist, a list of items was created to be subjected to the evaluation of 5 "peers" and 6 "experts".

#### Participants

In Mars 2020, 10 questionnaires were distributed on a convenience sample. The patients were recruited among those present at the Department of Clinical Psychology at Fondazione IRCCS Istituto Nazionale dei Tumori in Milan in three established recruitment days; the professionals were chosen among the collaborators of Department of Clinical Psychology of the hospital and experts in psychometrics. All the participants having provided written informed consent.

#### Measures

26 items belonging to 4 underlying theoretical dimensions were created: (1) Concern for your own health; (2) Emotional understanding; (3) Communication of emotions; (4) Feeling the same as others. For each item, "peers" and "experts" were asked to rate three aspects on a Likert scale ranged from 1 (= *not at all*) to 5 (= *very much*): comprehensiveness and representativeness. Furthermore, they were asked, if they deemed it necessary, to make changes to the items, to the delivery, and to report any aspect they wished.

#### Analysis

Interviews were transcribed verbatim. In order to maintain anonymity, identity references were modified in the transcript. The interviews were analyzed by two independent researchers [SA and LG] through a content analysis [31] using a pencil and paper modality. Reporting has been guided by the Consolidated Criteria for Reporting Qualitative Research (COREQ) checklist. All the contents that emerged from the interviews and in line with the aim were transformed into items and subjected to the judgment of "peers" and "experts". For each item, the content validity index (CVI) was calculated individually for the two investigated aspects (comprehensiveness and representativeness) and overall. To be considered satisfactory, values must be between 0.80 and 1.00 [32]. All comments made by "peers" and "experts" were discussed by two researchers [SA and LG] jointly, in order to accept or not the proposed changes.

### Results

#### Participants

A convenience sample of 4 patients (“peers”; 80% of compliance rate) and 6 professionals (“experts”; 100% of compliance rate) answered the questionnaire. The characteristics of the participants are shown in Table 1.

**Table 1:** Characteristics of “peers” and “experts”

	Peers (n = 4)		Experts (n = 6)	
	n	%	n	%
*Gender*				
Male	2	50	1	16.7
Female	2	50	5	83.3
*Level of education*				
Degree	4	100	1	16.7
Master’s degree	0	0	4	66.6
PhD	0	0	1	16.7
*Profession*				
Employee	1	25	0	
Doctor	1	25	0	
Retired	2	50	0	
Psychologist	0		1	16.7
Psychotherapist	0		4	66.7
Psychometrist researcher	0		1	16.7
Age (y.o.)		M *(SD)* Range		M *(SD)* Range
		57.00 *(14.09)* 33–66		51.66 *(15.26)* 31–72

#### Qualitative analysis

The results of the interviews with cancer patients highlighted four areas worthy of exploration: (1) the perception of cancer patients of being less afraid of the pandemic than the general population, as sooner or later a vaccine for CoViD-19 will be found, while for cancer it will be more difficult. At the same time, however, there is the concern of cancer patients that CoViD-19 could affect their treatment or assistance process of the cancer disease, as many visits and surgeries have been postponed (Concern for your own health); (2) the feeling of being more understood by people, because all human beings (sick and healthy) are now united by the feeling of unpredictability of the situation (Emotional understanding); (3) the feeling of being able to express one's emotions more easily, because during the pandemic it is more "legitimate" to talk about fears, anxieties and fatigue (Communication of emotions); (4) the perception of "feeling all in the same boat”, because the state of things caused by CoViD-19 has leveled some differences with other people that cancer patients normally feel (Feeling the same as others).

#### Content validity

As shown in Table 2, all items considered except item 19 are between 0.80 and 1.00. All items are visible in Additional file 1: Appendix 2. Since item 19 presents problems of comprehensibility rather than representativeness, it has been reformulated to be more understandable.

**Table 2:** Results of the validity of content carried out by "peers" and "experts"

	Mean comprehensiveness	Mean representativeness	Total mean	Content validity
*Concern for your own health*				
Item1	4.20	4.80	4.50	0.88
Item2	4.60	4.80	4.70	0.93
Item3	4.40	4.60	4.50	0.88
Item4	4.60	4.80	4.70	0.93
Item5	4.40	4.30	4.35	0.84
Item6	4.70	4.50	4.60	0.90
Item7	4.40	4.40	4.40	0.85
Item8	4.40	4.60	4.50	0.88
*Emotional understanding*				
Item9	4.70	4.80	4.75	0.94
Item10	4.70	4.60	4.65	0.91
Item11	4.30	4.20	4.25	0.81
Item12	4.60	4.70	4.65	0.91
Item13	4.40	4.50	4.45	0.86
Item14	4.70	4.70	4.70	0.93
*Communication of emotions*				
Item15	4.60	4.30	4.45	0.86
Item16	4.60	4.40	4.50	0.88
Item17	4.80	4.60	4.70	0.93
Item18	4.70	4.80	4.75	0.94
Item19	4.10	4.20	4.15	0.79
*Feeling the same as others*				
Item20	4.70	4.70	4.70	0.93
Item21	4.70	4.80	4.75	0.94
Item22	4.80	4.50	4.65	0.91
Item23	4.80	4.20	4.50	0.88
Item24	4.70	4.40	4.55	0.89
Item25	4.70	4.00	4.35	0.84
Item26	4.80	4.20	4.50	0.88

## Phase II

### Aim

Phase II aims to explore the factorial structure of the items and the theoretical dimensions that emerged from Phase I.

### Method

#### Participants and procedures

In the period between November 2020 and May 2021, we administered the questionnaire containing the 26 items that emerged from Phase I both in print and online format. The first was administered at the entrance of the hospital consecutively in three different weeks. 80 questionnaires were distributed. The online questionnaires were sent via link to all the cancer patients cared by 3 associations active nationally. All the participants having provided written informed consent.

#### Statistical methods

*Normality distribution.* In order to test the normality distribution of the item, Means (M), Standard Deviations (SD), Asymmetry, and Kurtosis were calculated. Following Darren and Mallery [27], item must have values beyond − 2/ + 2. We also calculated the Shapiro–Wilk test to confirm the normality of distribution of the items.

*Exploratory Factorial Analysis (EFA).* Preliminarily, to verify homoscedasticity, the Bartlett test must be calculated, which must be statistically significant. The Kaiser–Meyer–Olkin was also used to measure sampling adequacy.

Gerbing and Hamilton [33] suggest that EFA can be used prior to any analysis technique to confirm hypotheses on data structure. In order to reach a parsimonious solution, we performed a series of EFA on a first sample. We used Principal Axis Factoring with Oblimin Rotation that is the extraction method most widely used in the literature [34]. EFA was carried out using SPSS software V. 21.0.

### Results

#### Participants

215 patients answered the questionnaire. Among them, 31.2% completed the questionnaire in paper format (83.75% response rate). 8.6% are male, with a mean age of 58.83 years (range 18–85; SD = 12.37); 14.1% had an elementary or middle school diploma, 52.2% a high school diploma, 31.2% a degree (three or five years), 2.4% answered "other". 31.3% declared that they were patients exclusively by our hospital, the others stated that they were followed in other Italian hospitals, too.

#### Descriptive statistics

As shown in Table 3, both skewness and kurtosis for all the items fell between − 2/ + 2. Shapiro–Wilk test was statistically significant (*p* < 0.001) for all items, demonstrating the normality of the distribution.

**Table 3:** Mean, SD, skewness and kurtosis for all the Items

	Mean (range 1–4)	SD	Skewness	Kurtosis
Item1	2.99	.876	− .686	− .087
Item2	2.78	1.007	− .276	− 1.045
Item3	1.84	.935	.886	− .179
Item4	2.62	1.027	− .096	− 1.133
Item5	3.16	.898	− .855	− .085
Item6	3.08	1.004	− .710	− .713
Item7	2.68	.992	− .145	− 1.039
Item8	2.51	.994	.003	− 1.035
Item9	2.35	.956	.130	− .926
Item10	2.53	.970	− .028	− .964
Item11	2.33	.854	.019	− .692
Item12	2.37	.882	.076	− .715
Item13	2.51	.914	− .199	− .790
Item14	2.30	.905	.179	− .756
Item15	2.49	.994	.028	− 1.033
Item16	2.55	.986	− .116	− .997
Item17	2.55	.996	− .068	− 1.035
Item18	2.69	.967	− .258	− .883
Item19	2.87	1.001	− .475	− .848
Item20	2.43	.928	.084	− .831
Item21	2.41	.966	.045	− .967
Item22	2.34	.994	.130	− 1.042
Item23	2.17	.887	.353	− .600
Item24	2.26	.903	.277	− .677
Item25	2.56	.946	− .191	− .858
Item26	2.49	1.024	− .002	− 1.120

#### Exploratory factor analyses (EFA)

A series of EFAs were performed in order to reduce the set of items to a smaller, more parsimonious set, and to identify the number of factors to be retained. The final solution consists of 14 items that saturate three different dimensions, for a total explained variance of 64.58%.

The Kaiser–Meyer–Olkin Measure was found to be above 0.7 (= 0.89), to indicate that the sample is sufficient to perform the EFA. Bartlett's test was statistically significant, χ^2^ (325) = 2841.478, *p* < 0.001, which demonstrated the presence of homoscedasticity. All communalities of items had satisfactory values (between 0.38 and 0.86; see Table 4).

**Table 4:** Communalities

	Initial	Extraction
Item9	.547	.537
Item10	.593	.631
Item11	.559	.544
Item12	.571	.606
Item15	.793	.826
Item16	.832	.841
Item17	.832	.863
Item18	.650	.679
Item20	.600	.598
Item21	.802	.792
Item22	.803	.782
Item23	.514	.551
Item24	.427	.410
Item26	.388	.380

Table 5 shows the saturation pattern matrix of the 14 items on the three factors that emerged. All items clearly saturate a single factor, with values between 0.526 and 0.872. Factors 1 and 2 have Pearson's *r* correlation of 0.39, factor 1 and 3 of 0.56, factors 2 and 3 0.44.

**Table 5:** Pattern matrix of EFA

	Factor		
	Factor 1 Communication of emotions	Factor 2 Feeling the same as others	Factor 3 Emotional understanding
Item17	**.848**	− .036	.155
Item16	**.835**	.074	.083
Item18	**.793**	.047	.019
Item15	**.734**	.032	.244
Item22	.153	**.872**	− .132
Item21	.232	**.830**	− .117
Item23	− .193	**.767**	.077
Item20	.271	**.649**	− .042
Item24	− .116	**.618**	.130
Item26	.005	**.526**	.161
Item10	.043	− .031	**.783**
Item9	.007	.017	**.721**
Item12	.143	.076	**.650**
Item11	.202	.110	**.545**

The values that saturate a factor are shown in bold

## Phase III

### Aim

Phase III aims to verify the factorial structure that emerged from Phase II.

### Method

#### Participants and procedures

In October 2021, we administered the questionnaire containing the 14 items that emerged from Phase II in print format at the entrance of our hospital and in two different Department (Psychology and Radiology). One hundred forty paper questionnaires were distributed. All the participants having provided written informed consent.

#### Data analysis

*Confirmatory Factor Analyses (CFA).* We performed a Confimatory Factor Analyses (CFA) on a different sample of Phase II. The fit of the model was evaluated considering the values for acceptable absolute and relative fit indices. The selection of these indices was based on their statistical power and widespread use in Structural Equation Modelling. As indicative of absolute fit, we considered the values of the Standardized Chi-square (χ2/df < 5) and the Root Mean Square Error of Approximation (RMSEA < 0.08). As a relative fit index, we used the values of the Comparative fit index (CFI > 0.90) [35–37]. CFA was carried out using AMOS software V. 23.0.

*Comparative model.* As further confirmation of the dimensionality of the factorial structure, we wanted to test one alternative model that contemplated only one latent variable to which all the items appertain, with no distinction among dimensions. The underlying hypothesis is that all the items will saturate one factor (we can call it “Emotion during the CoViD pandemic”), so that there isn’t the distinction between the three latent factors that emerged in Phase II.

### Results

#### Participants

116 patients answered to the questionnaire (82.85% response rate). 34.5% are male, with a mean age of 61.37 years (range 20–89; SD = 15.61); 20.6% had an elementary or middle school diploma, 42.0% a high school diploma, 36.6% a degree (three or five years), 0.9% (1 person) answered "other". 57.1% said they went to the hospital for a check-up, 28.6% for therapy, 5.4% for a consultation, 8.9% answered "other" (e.g. surgery or for several reasons at the same time).

#### Confirmatory factory analysis (CFA)

**Fig. 1:**
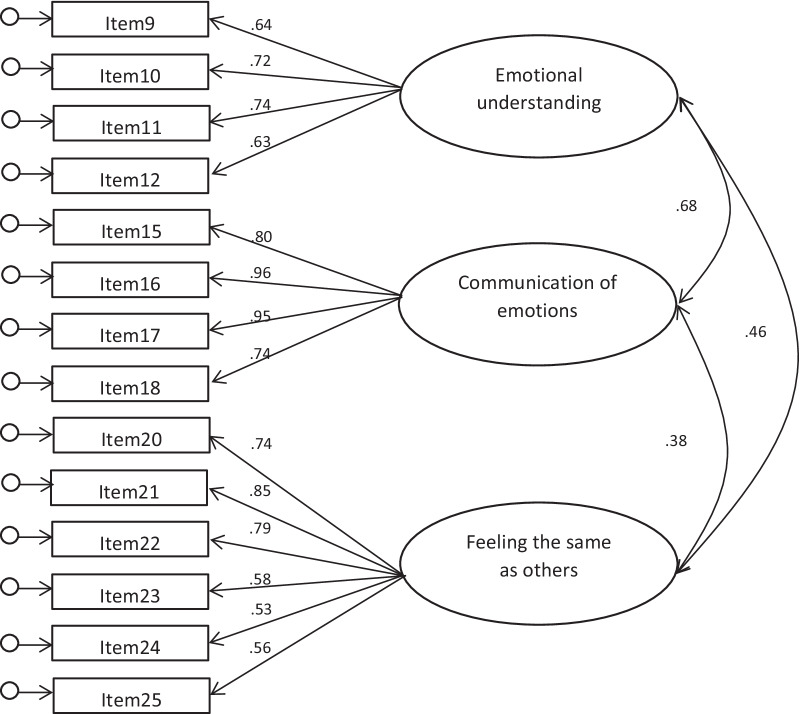
Results of CFA. *Notes* All factor loadings are statistically significant at *p* ≤ .01

The results of the CFA on the fourteen-item three-factor model showed that it achieved satisfactory fit indices. Indices of absolute fit are acceptable: χ^2^(74) = 127,79, *p* = 0.001, χ^2^/*df* = 1.72. RMSEA also is acceptable, = 0.079 (0.055-0.101). Relative fit index is good: CFI = 0.94. Figure 1 show results of CFA paths.

**Fig. 2:**
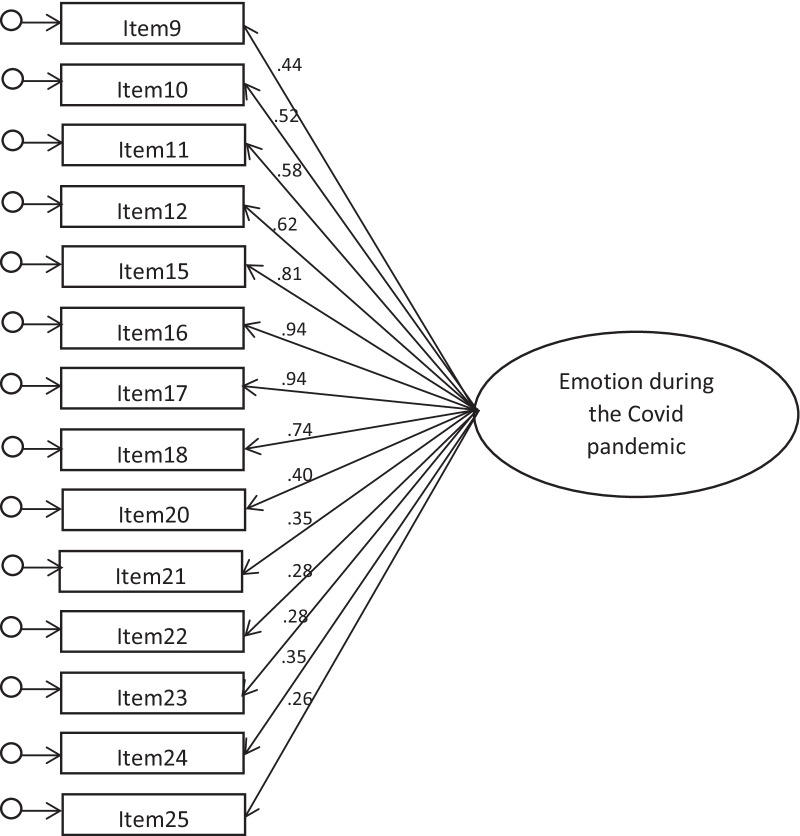
Alternative CFA model with one latent factor. Notes: All factor loadings are statistically significant at *p* ≤ .01

We tested one alternative model with only one latent variable to which all the items appertain, with no distinction between dimensions (Fig. 2). This model produced limited fit indexes: χ^2^(77) = 418,86, *p* < 0.001. χ^2^/*df* = 5.43; CFI = 0.63, RMSEA = 0.191 (0.173–0.209). Therefore, the hypothesis was not confirmed.

## Discussion

The present work aimed to begin the construction and initial validation of a questionnaire to investigate the emotions of cancer patients during the pandemic. Although numerous tools have been recently built aimed at the general population, to date there are no known questionnaires designed for detecting emotion in the cancer population.

Phase I highlighted the convergence between the "clinical feel" of the psycho-oncologist who work at the Fondazione IRCCS Istituto Nazionale dei Tumori in Milan, the quotations of the patients and the opinion of "peers" and "experts", giving reason to continue the research in the following phases. The results highlighted by Phase II and Phase III converge on three dimensions of the four hypothesized on a theoretical level: “Emotional understanding”, “Communication of emotions”, “Feeling the same as others”. Although some researches highlights the unfavorable impact of CoViD-19 which has caused disruption of treatment pathways and screening interventions [11, 13, 14], the theoretical dimension Concern for your own health is not confirmed. It is possible that adequate item were not chosen, or that they were not sufficiently well formulated, despite the good feedback provided by “peers” and “experts” in Phase I. Another interpretation is that this dimension is not "strong" enough for the target population of interest. The other three dimensions are instead robust and constant in the phases II and III of the survey. The feeling of belonging of cancer patients with the rest of the community (that we called Feeling the same as others) was also highlighted by the study of Schellekens & van der Lee [23].

The result of this study is even stronger if we consider that the three phases of the research were conducted in three different periods of the pandemic (phase I: at the beginning of the pandemic; phase II during a “red zone” period; phase III during a “yellow zone” period). The PEQ-CP has proved reliable despite the emotions and the experiences of cancer patients may have undergone variations due to the different levels of restrictions that have been taken by the authorities over time.

This work has some limitations that need to be considered. First, Phase II did not provide for the recruitment of cancer patients on a national basis, but only within a single center. Some differences in terms of intensity of emotions may be present based on the different limitations that the authorities have imposed on a regional basis. Secondly, as mentioned in the objectives, this work aims to start a process of construction and validation of a questionnaire. However, the validation has only started and further steps are necessary, first of all the convergent and discriminant validity. However, it is good to specify that to date there are no known questionnaires that have similar purposes to ours and in the same population.

Future research on larger population samples may or may not confirm the results obtained from this study. Subsequent research steps could be the measurement of the structural invariance between different tumor locations in cancer patients (e.g. breast, colon, lung cancer, etc.) to verify the strength of the factorial structure. Furthermore, it would be interesting to be able to monitor the constructs investigated with this questionnaire over time and verify correlating changes with some symptoms that are accentuated by a pandemic situation such as anxiety and depression.

One of the merits of this work is to have taken into consideration not only the negative or pathological aspects of the effects of CoViD-19 on cancer patients, but to have also considered the salutogenic aspects [26], such as resilience. Having a tool available to analyze these aspects is important both from a research and a clinical point of view, because it allows you to look at the phenomenon of the impact of CoViD-19 in this population from a different point of view.

## Supplementary Information

**Additional file1**: **Appendix 1**. Chronogram of the research phases in the development of the pandemic. **Appendix 2**. Italian and English version of the Pandemic Emotions Questionnaire in Cancer Patients (PEQ-CP).

## Data Availability

The datasets used and/or analysed during the current study are available from the corresponding author on reasonable request.

## Abbreviations

PEQ-CP: Pandemic emotions questionnaire in cancer patients
CoViD: Severe acute respiratory syndrome coronavirus 2
EFA: Exploratory factorial analysis
CFA: Confirmatory factor analyses
FCV: Fear of CoViD-19 scale
CAS: Coronavirus anxiety scale
SD: Standard deviation
M: Mean
RMSEA: Root mean square error of approximation
CFI: Comparative fit index
