# Supplementary material for: Construction process and development stages of pandemic emotions questionnaire in cancer patients (PEQ-CP)
Source: BMC Psychol. 2022 Sep 27;10:226. doi: 10.1186/s40359-022-00930-5 (PMC9514172; doi:10.1186/s40359-022-00930-5)
Supplement: Supplementary file 1 — Additional file1: Appendix 1. Chronogram of the research phases in the development of the pandemic. Appendix 2. Italian and English version of the Pandemic Emotions Questionnaire in Cancer Patients (PEQ-CP). [file 40359_2022_930_MOESM1_ESM.docx]

**Appendix 1**

|  | **Aim** | **Months** | **Covid Phase** |
| --- | --- | --- | --- |
| **Phase I** | Phenomenom survey and patients’ interviews | February 2020 | Warning of the first cases of Covid in Italy |
|  | Validity of content | March 2020 | WHO declares the state of pandemic |
| **Phase II** | Explorative factor analyses (EFA) | Since November 2020 to May 2021 | Begining of the second wave in Europe (lockdown and “red zone” in Lombardy) |
| **Phase III** | Confirmatory factor analyses (CFA) | October 2021 | Rising of the curve of the infection and state of emergency extended to 31st December 2021 (“yellow zone” in Lombardy) |

**Appendix 2**

| **Italian version** | **English version** |
| --- | --- |
| 1. Sono preoccupato/a di essere contagiato/a dal Covid-19 | 1. I’m worried about being infected by Covid-19 |
| 1. Sono preoccupato/a per i ritardi che l’emergenza Covid-19 sta provocando nel percorso di cura della mia malattia oncologica (controlli, interruzione dei nuovi protocolli di cura, ecc.) | 2. I’m worried about the delays that Covid-19 emergency is causing in the course of treatment of my oncological disease (check up, stop of new treatment protocols, etc.) |
| 1. Tendo a sottovalutare il rischio di venire contagiato/a dal Covid-19 | 3. I tend to underestimate the risk of infection by Covid-19 |
| 1. Sono più preoccupato/a per la mia malattia oncologica che di venire contagiato dal Covid-19 | 4. I am more worried about my oncological disease than about being infected by Covid-19 |
| 1. Sono fiducioso/a sulla scoperta di un vaccino per il Covid-19 | 5. I’m confident about the discovery of a vaccine for Covid-19 |
| 1. É più faticoso effettuare le cure oncologiche in ospedale senza familiari e amici | 6. It’s harder to make oncological treatments in hospital without relatives and friends |
| 1. É più difficile distrarmi dalle preoccupazioni legate alla mia malattia oncologica | 7. It’s harder to distract myself from worries connected to my oncological disease |
| 1. Le persone che mi sono vicine hanno più paura di venire contagiate dal Covid-19 rispetto a me | 8. People close to me are more afraid than me to be infected by Covid-19 |
| 1. Mi sento più compreso/a di prima dalle persone che mi circondano | 9. I feel myself more understood than before by people around me |
| 1. Sento che le persone accanto a me hanno più attenzione nei miei confronti | 10. I feel that people around me are more careful with me |
| 1. Sento che le persone accanto a me sono più capaci di immedesimarsi nella mia situazione | 11. I feel that people around me are more able to empathize with my condition |
| 1. Mi sento sollevato/a perché gli altri sono più comprensivi e vicini emotivamente | 12. I feel relieved because the others are more understanding and emotionally closer |
| 1. Sento i medici più sensibili alla mia condizione emotiva | 13. I feel the doctors more sensitive about my emotional condition |
| 1. Sento che le persone comprendono di più cosa significa essere malati oncologici | 14. I feel that people understand more what it means to be an oncological patient |
| 1. Sento di poter parlare di più delle mie paure con chi mi è vicino/a | 15. I feel that I can talk more about my fears with people close to me |
| 1. Mi sento più libero/a di manifestare i miei sentimenti con chi mi è vicino/a | 16. I feel more free to express my feelings with people close to me |
| 1. Sento di poter comunicare maggiormente le mie emozioni a chi mi è vicino/a | 17. I feel I can comunicate more my emotions to people close to me |
| 1. Sento di poter parlare delle mie paure | 18. I feel I can talk about my fears |
| 1. Sento che “siamo tutti sulla stessa barca” | 19. I feel that “we are all in the same boat” |
| 1. Mi sento meno solo/a di prima perché tutti stiamo vivendo la stessa situazione | 20. I feel less alone than before because everyone is living the same situation |
| 1. Mi sento più simile agli altri | 21. I feel more similar to the others |
| 1. Sento gli altri più simili a me | 22. I feel others more similar to me |
| 1. Mi sento più rilassato/a perché anche gli altri hanno dovuto ridurre le loro attività | 23. I feel more relaxed because the others have had to reduce their activities, too |
| 1. Sento che la mia malattia oncologica fa meno paura agli altri | 24. I feel that my oncological disease is less scary for others |
| 1. La paura è un sentimento che stiamo provando tutti | 25. Fear is a feeling that everybody is experiencing |
| 1. Vedere che tutti portano la mascherina mi fa sentire più simile agli altri | 26. To see that everyone wears a mask makes me feel more similar to the others |
